# Supplementary material for: SMA20/PMIS2 Is a Rapidly Evolving Sperm Membrane Alloantigen with Possible Species-Divergent Function in Fertilization
Source: Int J Mol Sci. 2024 Mar 25;25(7):3652. doi: 10.3390/ijms25073652 (PMC11011635; doi:10.3390/ijms25073652)
Supplement: Supplementary file 1 [file ijms-25-03652-s001.zip › ijms-2872672-supplementary.pptx]

## Slide 1
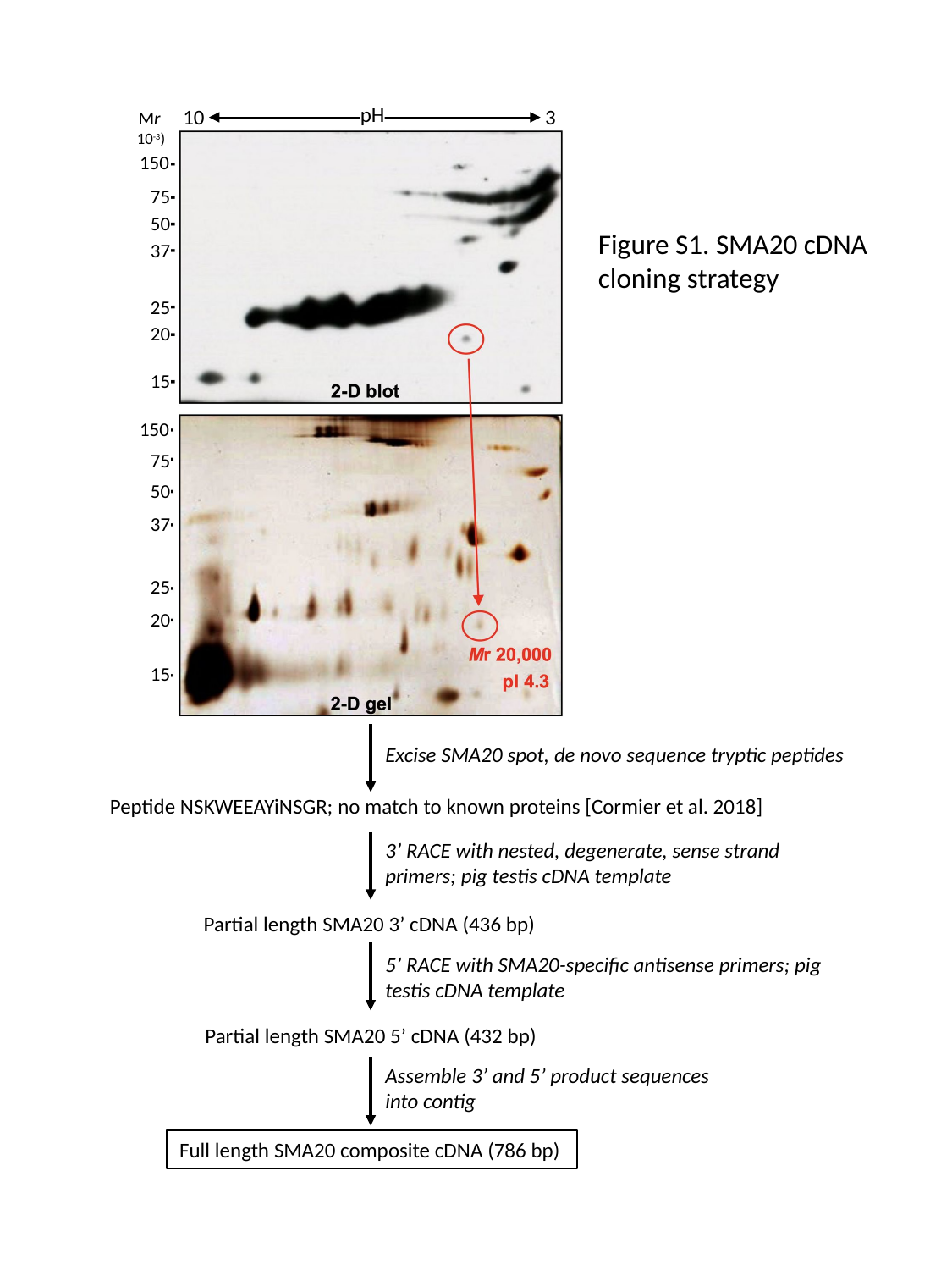

pH
10
3
150
75
50
Figure S1. SMA20 cDNA cloning strategy
37
25
20
15
150
75
50
37
25
20
15
Excise SMA20 spot, de novo sequence tryptic peptides
Peptide NSKWEEAYiNSGR; no match to known proteins [Cormier et al. 2018]
3’ RACE with nested, degenerate, sense strand primers; pig testis cDNA template
Partial length SMA20 3’ cDNA (436 bp)
5’ RACE with SMA20-specific antisense primers; pig testis cDNA template
Partial length SMA20 5’ cDNA (432 bp)
Assemble 3’ and 5’ product sequences into contig
Full length SMA20 composite cDNA (786 bp)

## Slide 2
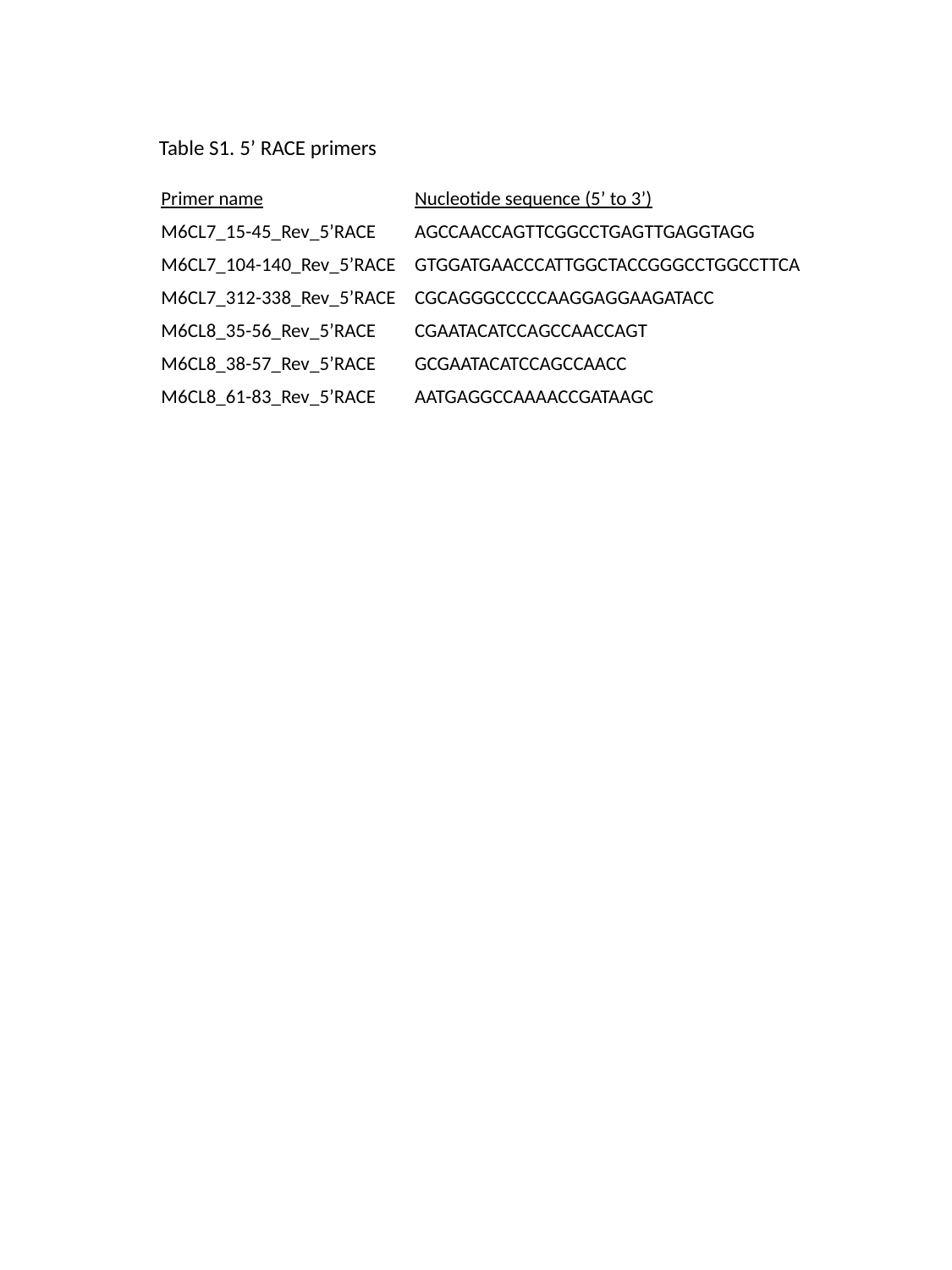

Table S1. 5’ RACE primers
| Primer name | Nucleotide sequence (5’ to 3’) |
| --- | --- |
| M6CL7\_15-45\_Rev\_5’RACE | AGCCAACCAGTTCGGCCTGAGTTGAGGTAGG |
| M6CL7\_104-140\_Rev\_5’RACE | GTGGATGAACCCATTGGCTACCGGGCCTGGCCTTCA |
| M6CL7\_312-338\_Rev\_5’RACE | CGCAGGGCCCCCAAGGAGGAAGATACC |
| M6CL8\_35-56\_Rev\_5’RACE | CGAATACATCCAGCCAACCAGT |
| M6CL8\_38-57\_Rev\_5’RACE | GCGAATACATCCAGCCAACC |
| M6CL8\_61-83\_Rev\_5’RACE | AATGAGGCCAAAACCGATAAGC |
